# Wound contraction effects and antibacterial properties of *Tualang *honey on full-thickness burn wounds in rats in comparison to hydrofibre

**DOI:** 10.1186/1472-6882-10-48

**Published:** 2010-09-03

**Authors:** Yan-Teng Khoo, Ahmad Sukari Halim, Kirnpal-Kaur B Singh, Noor-Ayunie Mohamad

**Affiliations:** 1 Reconstructive Sciences Unit, School of Medical Sciences, Universiti Sains Malaysia, Malaysia; 2 Department of Medical Microbiology & Parasitology, School of Medical Sciences, Universiti Sains Malaysia, Malaysia

## Abstract

**Background:**

Full-thickness burn wounds require excision and skin grafting. Multiple surgical procedures are inevitable in managing moderate to severe full-thickness burns. Wound bed preparations prior to surgery are necessary in order to prevent wound infection and promote wound healing. Honey can be used to treat burn wounds. However, not all the honey is the same. This study aims to evaluate the wound contraction and antibacterial properties of locally-produced *Tualang *honey on managing full-thickness burn wounds *in vivo*.

**Methods:**

Thirty-six female *Sprague Dawley *rats were randomly divided into three groups. Under anaesthesia, three full-thickness burn wounds were created on the dorsum of the rats. The full-thickness burn wounds were inoculated with a specific organism (10^4^), namely *Pseudomonas aeruginosa *(n = 12), *Klebsiella pneumoniae *(n = 12), or *Acinetobacter baumannii *(n = 12). The three burn wounds were dressed with *Tualang *honey, hydrofibre and hydrofibre silver respectively. Swab samples were obtained every 3 days (day 3, 6, 9, 12, 15, 18 and 21) for quantitative and semi-quantitative microbiological analyses. Clinical assessments, including observations concerning the appearance and wound size, were measured at the same time.

**Results:**

There was a rapid 32.26% reduction in wound size by day 6 (*p *= 0.008) in the *Tualang *honey-treated wounds, and 49.27% by day 15 (*p *= 0.005). The wounds remained smaller by day 18 (*p *< 0.032). *Tualang *honey-treated rats demonstrated a reduction in bacterial growth in *Pseudomonas aeruginosa *inoculated wounds (*p *= 0.005). However, hydrofibre silver and hydrofibre-treated wounds are superior to honey-treated wounds with *Acinetobacter baumannii *(*p *= 0.035). There was no statistical significant of antibacterial property in *Klebsiella pneumonia *inoculated wounds.

**Conclusions:**

*Tualang *honey has better results with regards to its control of *Pseudomonas aeruginosa *and its wound contraction effects on full-thickness burn wound *in vivo*.

## Background

Full-thickness burn wounds will not heal spontaneously, and inevitably need excision. Closure of the resultant wound is required in order to reduce the risks of invasive infection, which can result in systemic sepsis. In a majority of patients with full-thickness burns, early excision and immediate autografting is recommended [1]. In addition, it has been shown that full-thickness burn wounds are more commonly associated with infection than other tissue defects [2]. Infection of full-thickness burn wounds is frequently caused by bacterial organisms; the most common are *Pseudomonas aeruginosa, Klebsiella pneumoniae *and *Acinetobacter baumannii*. Given these problems, development of an alternative technique or dressing to reduce burn wound infection is of importance.

Honey can be a remedy for wound care. It is the oldest remedy for treating wounds, and dates back to the sixth century AD [3]. The ancient Egyptians used honey in a grease-honey-lint dressing to put on infected wounds. This traditional cure was displaced in the 1940 s, before bacteria were discovered to be the cause of infection and with the discovery of antibiotics. It has recently been rediscovered by the medical profession, particularly where conventional modern therapeutic agents fail and with the trend of antibiotic-resistant wounds.

The current prevalence of antibiotic-resistant microbial species has led to a re-evaluation of the therapeutic use of ancient remedies such as honey. Honey has been reported to aid in wound healing, as it has special antibacterial and antibiotic properties [4]. More recently, honey has been reported to have an inhibitory effect on approximately 60 species of bacteria, including aerobes and anaerobes, gram-positive and gram-negative bacteria [5]. An antifungal action has also been observed for some yeast and species of *Aspergillus *and *Penicillium*, as well as all the common dermatophytes [5,6]. There are now many published reports describing the effectiveness of honey in rapidly clearing infections from wounds and protecting wounds from becoming infected [5,7-10]. Thus, it provides a moist healing environment without the risk of bacterial growth occurring, and with no adverse effects to slow the healing process. An infected wound will not heal unless bacteria are eliminated, as bacteria stimulate the inflammatory response, which can hinder the process of wound healing.

A honey dressing can undoubtedly be used on many types of wounds. Hence, previous study has produced an ideal wound dressing incorporating calcium alginate fibres and active *Manuka *honey [11]. It is believed that this alginate dressing offers antibacterial barrier protection and encourages wound healing. Many researchers have established the potency of *Manuka *honey to heal infected wounds and prevent bacterial infection [5,11]. However, not all honey is the same.

*Tualang *honey is a type of honey produced locally in Malaysia. The major components of *Tualang *honey are furfural derivatives such as 5-(hydroxymethyl)-furfural or HMF (25.4-185.6 mg/kg), furfural (46.9-58.5 mg/kg), 2-furylmethylketone (0.2-0.9 mg/kg), 5-methyl furfural (2.2-3.6 mg/kg) and fatty acids such as palmitic acid (341.0-531.4 mg/kg), ethyl linoleate (2.0-46.7 mg/kg) and ethyl oleate (1.6-19.1 mg/kg) [12]. A local study about the volatile components of *Tualang *honey has revealed 35 volatile compounds compared to other studies by Lušić and Odeh [13]. Odeh identified 30 compounds in Palestinian honey and Lušić identified 37 compounds from lime tree honey samples using solid phase microextraction (SPME) [14,15]. *Tualang *honey shares similar compounds, but it contains compounds that were not documented in literature before. They are terpenes and phynylethanal, which have significant results on microbiological activity. Terpene is a type of phytochemicals that contributes to the antimicrobial activity of honey [16]. Phenylethanal is an enzyme, reported to have some bactericidal effects.

Nuriza had performed an *in vitro *experiment on antibacterial activity of 5 types of Malaysian honey: *Tualang, Hutan, Gelang, Pucuk Daun *and *Ee Feng Gu*. Significant variation in composition of the honeys was noted. *Tualang, Pucuk Daun *and *Ee Feng Gu *honey had significant antibacterial activity against *S.typhi, Staphy.aureus, S.Sonnie *and *E.coli in vitro *[17].

*Clostridium botulinum *survives in honey, and therefore wound is at risk of botulism [18]. It is therefore recommended the honey to be sterilized by gamma irradiation at a dose of 25 kGy. Study has shown that radiation at 25 and 50 kGy do not affect the quality of honey used [19].

The good wound healing properties of Malaysian honey is based on the previous studies of positive effect of honey on animal study, whereby the efficacy of using honey dressing was established [20,21]. To our knowledge, there is no published report on animal study testing specifically the efficacy of *Tualang *honey compared to hydrofibre silver on full thickness burn wound. This study performed a direct comparison with the most commonly used modern dressing in clinical practice. This study used *Tualang *honey which has not been studied using randomized method on full thickness burn wound; which represents a robust study design. Further research is needed to optimize the effective use of this agent in clinical practice. Nevertheless, its potency on wound healing and the effectiveness of its antibacterial properties can be studied *in vivo *before clinical use. This study was conducted to evaluate the wound healing and antibacterial properties of *Tualang *honey in full-thickness burn wounds in a *Sprague Dawley *rat model.

## Methods

### Study design

A randomized method was used for this animal study. This study was performed in the Laboratory of Animal Research Unit, Universiti Sains Malaysia. All the *Sprague Dawley *rats in this study were purchased from the animal centre of Hospital Universiti Sains Malaysia. The sample size was estimated using the two proportions formula with an 80% confidence interval. The sample size calculation was performed using the Power and Sample Size Calculation system (PS), with a type 1 error of 0.025. A total of 36 rats were required for this study. The inclusion criteria included *Sprague Dawley *rats weighing between 250 and 350 grams.

### Experimental protocol

All *Sprague Dawley *rats received humane care in compliance with the 'Principles of Laboratory Animal Care' formulated by the American Society for Medical Research and the 'Guide for the Care and Use of Laboratory Animals' prepared by the American Academy of Sciences and published by the National Institute of Health, United States of America. This study was approved by the university's animal ethics committee, USM Health campus with reference number of USM/Animal Ethics Approval/2007/(34)(108).

The rats were housed individually in cages, and fed with free access to standard commercial rat food and water throughout the study. Thirty six rats was divided into 3 groups randomly i.e. 12 rats in each group (Group A, B & C).

*Tualang *honey certified by Federal Agricultural Marketing Authority (FAMA), Malaysia, was used for wound dressing. This honey was collected in February and March of year 2009. This honey was sterilized by γ-irradiation at a dose of 25 kGy. The samples were stored in sealed, Teflon-coated plastic vials with silicone septa (Alltech, Milano, Italy) in a cool place before use. Hydrofibre and hydrofibre silver dressings used are plain Aquacel and Aquacel silver respectively. These dressings are produced by ConvaTec Inc, Skillman NJ 08558.

### Anaesthesia and surgical protocol

On the day of wounding, the rats were placed in a ventral position and immobilized on their abdomen for the surgery. The dorsum of each rat was shaved. Immediately before the operation, the rats were anaesthetized with an intramuscular injection of 35.0 mg/kg Ketamine and 5.0 mg/kg Xylazine in the gluteal area. When fully anaesthetized, the shaved areas were cleaned with povidone iodine, alcohol and Hibiscrub^®^. The operation site was isolated with a sterile towel. Full-thickness burn wounds were created using sterile technique on the dorsum of the rats using hot metal heated with a burner at a temperature of 100°C for 30 seconds. A total of three full-thickness burn wounds were created on the dorsum of each rat. Each wound was 10 mm by 10 mm, 20 mm apart. The wound was inoculated with 10^4 ^colony forming unit (CFU) of one of three common skin wound contaminants, namely *Pseudomonas aeruginosa *(Group A, n = 12), *Klebsiella pneumonia *(Group B, n = 12) or *Acinetobacter baumannii *(Group C, n = 12). All the wounds in Group A (n = 12) were inoculated with *Pseudomonas aeruginosa*. All the wounds in Group B were inoculated with *Klebsiella pneumonia*. All the wounds in Group C were inoculated with *Acinetobacter baumannii*. All the rats (Group A, B & C) received honey dressing on the first burn wound, hydrofibre on the second wound, and hydrofibre silver on the last wound. A thin layer of pure undiluted *Tualang *honey (0.1 ml/cm^2^) was applied topically to the first burn wound and filled up the wound. It was then covered with plain gauze. Hydrofibre and hydrofibre silver were applied to the other two wounds. All the dressing materials were covered with plain gauze separately. All the wounds were reinforced with crepe bandage. Thus, each group of rats received three wound dressings (*Tualang *honey, hydrofibre, and hydrofibre silver).

### Post-surgery care and follow up

The animals were monitored immediately postoperatively for spontaneous breathing efforts and movement. After surgery, each animal was housed in an individual cage in a room and fed with standard rat diet and water, post-operative subcutaneous injection of morphine 0.02 mg/kg was given. All wounds were cleaned with normal saline and treatment reapplied every three days. All wounds were assessed clinically according to a scoring system (Table 1). The rats were then humanely euthanized with an intra-peritoneal injection of 5 mg phenobarbitone sodium.

**Table 1:** Dressing and wound evaluation system

Dressing Evaluation				
Flexibility	0	1	2	3
	(Not flexible)	(Minimal flexible)	(Moderate flexible)	(Flexible)
				
Adherence	0	1	2	3
	(Non-adherent)	(Minimal-adherent)	(Moderate-adherent)	(Good adherent)
				
Ease of removal	0	1	2	3
	(Very Difficult)	(Difficult)	(Easy)	(Very easy)
				
Fluid accumulation	0	1	2	3
	(Yes)	(Moderate)	(Minimal)	(No)

**Wound Evaluation**				

Dryness of wound area	1	2	3	
	Wet		Dry	
				
Exudation	1	2	3	
	Heavy exudates	Exudate	No exudates	
				
Wound Odour	1	2	3	
	Strong Odour	Mild Odour	Odourless	
				
Wound Contraction	1	2	3	
	Very Contracted	Contracted	Not Contracted	

### Evaluation of wound size

The wounds were subjected to evaluation every three days, i.e., on day 3, 6, 9, 12, 15, 18 and 21. Each wound was examined and photographs were taken after burn wound creation until healing was complete. Clinical assessments included observations concerning the appearance, and the wound size was measured using graph paper (mm^2^). The wound size was measured from the periphery of the wound.

### Microbiological examinations

Swabs were taken from the burn wound during each dressing change on day 3, 6, 9, 12, 15, 18 and 21. The collected swabs were immediately sent to the laboratory for testing.

In the quantitative count study, 2 ml of normal saline was added to each of the samples. The sample was vortexed thoroughly and a 10-fold serial dilution was performed. Eight hundred microliters of each sample dilution was spread onto Tryptic Soy Agar (TSA). Two replicates were carried out for each dilution, and the agar plates were incubated at 37°C for 24 hours. The colonies were counted, and results were tabulated [22].

In the semi-quantitative analysis, swabs from wounds were spread onto blood agar plates according to the method described by Henry [23]. After 24 hours incubation at 37°C, the blood agar plates were removed from the incubator and the growth of bacteria on each plate was scored as stated in Table 2.

**Table 2:** Semi-quantitative analysis scoring system for microbiological examinations

Score	Number of colonies in the streak area		
	
	**1**^**st **^**Quadrant**	**2**^**nd **^**Quadrant**	**3**^**rd **^**Quadrant**
1+	<10		

2+	<10	<5	

3+	>10	>5	<5

4+	>10	>5	>5

### Statistical analysis

All the data were examined statistically. Data entry and analysis were done using Statistical Package for Social Sciences (SPSS) version 12.0. The values were expressed as Mean and Standard Deviation. Repeated measurements of Analysis of Variance (ANOVA) were used to compare mean differences and numerical data between and within groups, and the level of statistical significance was set at 0.05.

Treatment groups were used as the independent grouping variable, and the wound contraction size (mm) and the quantitative and semi-quantitative count of micro-organisms were used as the dependent variable.

The association between the dressing material and microbiological analysis was examined by non-parametric t-test. The final model of wound size used Kruskal-Wallis test. The *p *value was significant, thus the model was fit. The main purpose of the model was to determine the interactions by using a two-way interactions test.

## Results

### Clinical examinations

*Tualang *honey dressing was more flexible, less adherent to the wound base, easier to peel off during dressing changes and caused less fluid accumulation in the wound.

**Figure 1:**
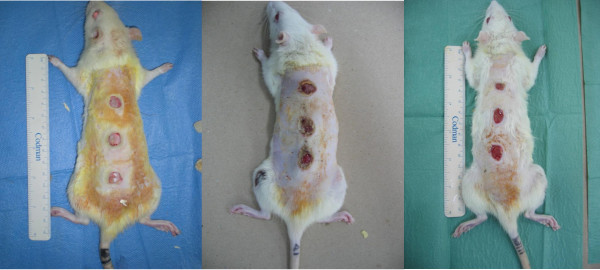
**In *Pseudomonas aeruginosa *inoculated groups, post-burn wounds on day 3; day 9 and day 15**.

**Figure 2:**
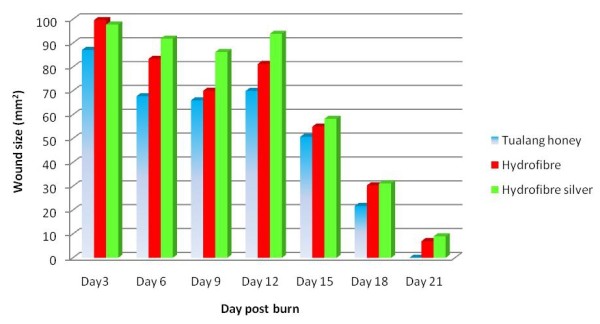
**Comparison of wound size among three different dressing materials on burn wounds over time**.

Comparison of the time course of post-burn wound sizes in the *Tualang *honey-treated wounds and hydrofibre silver-treated wounds showed that the mean wound size in the former group was statistically smaller. Wound size was found to be markedly reduced in the *Tualang *honey-treated wounds on day 3, 9 and 15 (Figure 1). The wounds showed a reduction in size of 12.86% by day 3 from the original 100 mm^2 ^in the *Tualang *honey-treated wounds (*p *= 0.010). They further decreased in size of 33.94% by day 9 post-burn respectively (Figure 2). In the hydrofibre silver-treated wounds, the reduction from the original wound size was only 2.20% on day 3 (*p *= 0.010). The wounds were reduced by 13.74% by day 9 (*p *= 0.003). Over time, the reduction of wound size was shown in Figure 2. To determine the long term effect of *Tualang *honey on burn wounds, the wound healing process was observed up to 21 days. On day 21, honey-treated wounds in *Pseudomonas aeruginosa *inoculated group and *Acinetobacter baumannii *inoculated groups healed completely. The remaining wounds in *Pseudomonas aeruginosa *inoculated group and *Acinetobacter baumannii *inoculated groups and all the wounds in *Klebsiella pneumonia *inoculated wounds did not healed completely.

### Microbiological examinations

**Figure 3:**
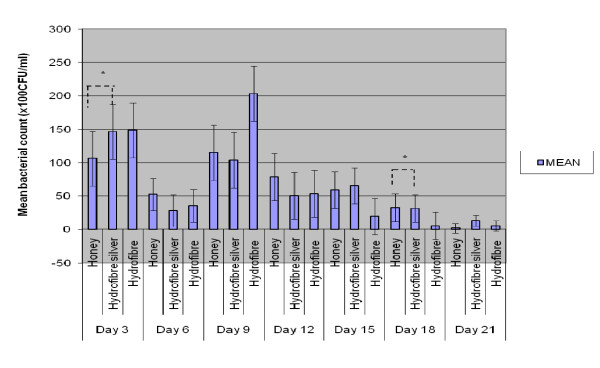
**Comparison of colony count (CFU) among 3 wound dressings over time in *Pseudomonas aeruginosa *wounds**. n = 36, * p < 0.05.

In the quantitative analysis, the *Tualang *honey-treated rats demonstrated a reduction in bacterial growth compared to hydrofibre silver and hydrofibre-treated wounds in *Pseudomonas aeruginosa *inoculated wounds (*p *= 0.005), with marked bacterial growth reductions on day 3, 12, 15, 18 and 21 of the experiment (Figure 3). However, hydrofibre silver and hydrofibre-treated wounds were superior to honey-treated wounds in *Acinetobacter baumannii *inoculated wounds (*p *= 0.035). There was no statistically significant difference between the three dressing materials used in *Klebsiella pneumonia *inoculated wounds.

The semi-quantitative examinations of the three organisms, *Pseudomonas aeruginosa, Klebsiella pneumoniae *and *Acinetobacter baumannii*, in the full-thickness burn wounds were performed in this study. In the *Pseudomonas aeruginosa *group, wounds treated with *Tualang *honey had decreased bacterial counts in the initial 6 days post-burn injury (day 3 and 6). By day 18 of the experiment, the bacterial growth was reduced to minimal in *Tualang *honey-treated and hydrofibre silver-treated wounds. There was no statistically significant difference in *Klebsiella pneumoniae *and *Acinetobacter baumannii *inoculated wounds.

## Discussions

Many reports have been published about the usefulness of honey in wound management [5,11,24,25]. Honey is commonly utilized to dress wounds, for instance in burn wounds and other infected wounds [26]. Treatment of fresh wounds with honey was noted to produce increased wound contraction and an increase in granulation tissues [27]. Not all the honeys are the same; different types of honey have been used in many animal experiments to show acceleration in wound healing and its antibacterial properties [17,19,28]. The utility of *Tualang *honey for dressing burn wounds was demonstrated in this study.

The efficiency and efficacy of the topical application of honey on burns have been reported by previous study [21]. Molan found that the application of *Manuka *honey accelerated healing in acute wounds, chronic ulcers, burns, MRSA infections and infected wounds [29]. It shortens the healing period of the wounds. Hence, it is used in this study to test its properties on full-thickness burn wounds in an animal model. A report revealed that a honey dressing enhances wound contraction in fresh wounds, which is one of the key features of wound healing [27]. It is clear from this experiment that the *in vivo *wound contraction of burn wounds dressed with *Tualang *honey was markedly greater than with the hydrofibre silver and hydrofibre dressing. It is especially true in the first 6 days post-burn injury, when marked wound contraction was observed. The area of the wound was smaller, and epithelialization from the periphery of the wound edge was increased. The wound healing process was accelerated in the first 2 weeks in *Tualang *honey-treated wounds as compared to hydrofibre silver-treated wounds, throughout our experimental study. This shows that *Tualang *honey has the effect of promoting wound contraction in full-thickness burn wounds.

In this study, we noticed that the scabs formed by the honey-treated wounds were thinner compared to the other two treatment groups. It is believed that this condition could be attributed to the moist environment created by the honey due to its viscosity and high sugar content. Thus, thinner scabs form smaller barriers for the epithelialization to occur, and further accelerate the healing process. This observation indicates that the maximum wound contraction was in the *Tualang *honey group. This result was obtained by applying 0.1 ml/cm^2 ^concentration of honey topically on the burn wounds. Our results suggest that *Tualang *honey applied topically to the burn wound accelerates the healing process. This will reduce the amount of autologous skin grafting necessary for full-thickness burn wound coverage, especially in minor burn.

After the creation of full-thickness burn wounds on *Sprague Dawley *rats, the *Tualang *honey was applied to the burned areas for 21 days, with treatment application every 3 days. Swab samples were collected at day 3, 6, 9, 12, 15, 18 and 21 after the burn wound was created. The amount of the specific organism was analyzed using both quantitative and semi-quantitative methods. Molan demonstrated that *Manuka *honey has antibacterial properties against the *Staphylococci *and *Pseudomonas aeruginosa *[29]. There is report that honey is able to inhibit growth of both Gram-negative and Gram-positive organisms [30]. It is believed that the antimicrobial properties maybe due to hyper-tonicity and low pH, as well as catalase in the honey, which inhibits the growth of microorganisms [6,28]. The effectiveness of honey as a dressing material can be attributed to its antimicrobial properties. The effectiveness of antibacterial properties of Malaysian *Tualang *honey was shown to be equivalent or better compared to *Manuka *honey *in vitro *[31]. Another study revealed that *Tualang *honey has comparable result for Gram-negative bacteria *in-vitro *[32]. The quantitative analysis of microbiological examination of this study has shown that *Tualang *honey-treated rats demonstrated a reduction in bacterial growth compared to hydrofibre silver and hydrofibre-treated wounds in *Pseudomonas aeruginosa *inoculated wounds (*p *= 0.005), with marked bacterial growth reductions. Semi-quantitative analysis of the *Pseudomonas aeruginosa *group showed that *Tualang *honey treated wounds had significant decreased bacterial counts in the initial 6 days post-burn injury. Both the quantitative and semi-quantitative analyses indicated that the *Pseudomonas aeruginosa *control by *Tualang *honey was superior compared with the other two dressing groups. *In vitro *study reported by Tan also showed that *Tualang *honey exhibit strong antibacterial activity against *Pseudomonas aeruginosa *[31]. This report showed the effectiveness of *Tualang *honey in controlling the proliferation of *Pseudomonas aeruginosa*. It can prevent burn wound infections in those *Pseudomonas aeruginosa *inoculated burn injury. The wound healing process will slow down if there is presence of underlying infection. The healing process will proceed orderly only when the infecting bacteria is cleared. This may further reduce the incidence of systemic sepsis and mortality. Previous studies reported that *Manuka *honey and honey pastures were effective in preventing the growth of *Pseudomonas *on the surface of wounds [33]. Local application of raw honey on infected wounds reduced the time for eradication of the bacterial infection due to *Klebsiella sp *[34]. However, in this study, the overall poor antibacterial activity of *Tualang *honey against *Klebsiella pneumoniae *was unexpected in light of previous reports. The difference in results may be due to the different types of honey used in the studies. Previous studies used variable concentrations of honey to test the eradication of bacterial infections, and the present study used pure *Tualang *honey to determine its efficacy against *Klebsiella pneumoniae*. It is likely due to the variation in the composition of *Tualang *honey used in the present study. Part of the explanation may be that *Klebsiella pneumoniae *is a Gram-negative encapsulated organism, which reduces the penetration of honey.

*Pseudomonas aeruginosa, Acinetobacter baumannii *and *Klebsiella pneumoniae *are common pathogens that can cause nosocomial infections in a hospital setting. A study on the rate of infections in burns showed that in 65% of fatal burn cases, septicaemia was the cause of death, and *Pseudomonas aeruginosa *and *Klebsiella sp*. were the most common organisms. These organisms have resistance to many antibiotics and have become the predominant agents of wound sepsis in hospitals [35]. This study shows inhibition of *Pseudomonas aeruginosa *by *Tualang *honey with a topical application at a concentration of 0.1 ml/cm^2^. This shows the efficacy of antibacterial factors present in *Tualang *honey. Therefore, *Tualang *honey plays an important role in controlling the growth of bacteria, especially *Pseudomonas aeruginosa*, which may further avoid the burn wound infections that lead to systemic sepsis. The quantitative and semi-quantitative analyses indicated and supported that the *Pseudomonas aeruginosa *control by *Tualang *honey was superior compared with the other treatment groups. The relative benefit needs to be evaluated in clinical trials.

## Conclusions

This experiment shows the positive effect of *Tualang *honey as a sound topical dressing for full-thickness burn wounds in an animal model. *Tualang *honey had better results with regard to its control of *Pseudomonas aeruginosa *infection and its wound contraction effects on burn wounds.

## Competing interests

The authors state that we have no competing interests. Each author has contributed original work to the manuscript and adhered to the ethical requirements as outlined by the journal.

## Authors' contributions

KYT carried out the animal experimental study, participated in the microbiological examination and drafted the manuscript. ASH designed the study and jointly drafted the manuscript. KKBS contributed to microbiological examination and analysis. MN and RAR contributed to the assistance for microbiological examinations. NAM contributed to laboratory work of histopathological examination. All authors read and approved the final manuscript.

## Pre-publication history

The pre-publication history for this paper can be accessed here:

http://www.biomedcentral.com/1472-6882/10/48/prepub

## References

[B1] Wang JD Early excision and one-stage grafting with full-thickness autologous skin in total deep burn of the face Zhonghua Zheng Xing Shao Shang Wai Ke Za Zhi 1987 3 103 105 3151575

[B2] Smith DJ Jr Thomson PD Garner WL Rodriguez JL Burn wounds: infection and healing American Journal of Surgery 1994 167 46S 48S 10.1016/0002-9610(94)90011-6 8109685

[B3] Golder W Propolis. The bee glue as presented by the Graeco-Roman literature Wurzbg Medizinhist Mitt 2004 23 133 145 15630803

[B4] Karayil S Deshpande SD Koppikar GV Effect of honey on multidrug resistant organisms and its synergistic action with three common antibiotics Journal Postgraduate Medicine 1998 44 93 96 10703581

[B5] Molan PC Re-introducing honey in the management of wounds and ulcers - theory and practice Ostomy Wound Manage 2002 48 28 40 12426450

[B6] Wilkinson JM Cavanagh HM Antibacterial activity of 13 honeys against Escherichia coli and Pseudomonas aeruginosa J Med Food 2005 8 100 103 10.1089/jmf.2005.8.100 15857217

[B7] Cooper RA Halas E Molan PC The efficacy of honey in inhibiting strains of Pseudomonas aeruginosa from infected burns J Burn Care Rehabil 2002 23 366 370 10.1097/00004630-200211000-00002 12432313

[B8] Allen KL Molan PC Reid GM A survey of the antibacterial activity of some New Zealand honeys J Pharmacol 1991 43 12 817 822 10.1111/j.2042-7158.1991.tb03186.x 1687577

[B9] Willix DJ Molan PC Harfoot CG A comparison of the sensitivity of wound-infecting species of bacteria to the antibacterial activity of manuka honey and other honey J Appl Bacteriol 1992 73 5 388 394 1447054 10.1111/j.1365-2672.1992.tb04993.x

[B10] Fruncillo RJ DiGregorio GJ The effect of thermal injury on drug metabolism in the rat J Trauma 1983 23 6 523 529 10.1097/00005373-198306000-00014 6864844

[B11] Jull A Walker N Parag V Molan PC Rodgers A Randomized clinical trial of honey-impregnated dressings for venous leg ulcers Br J Surg 2008 95 175 182 10.1002/bjs.6059 18161896

[B12] Man Che Nin Mohamed Mahaneem Sulaiman Siti Amrah The chemical compositions of Tualang honey2nd International Conference on the Medicinal Use of Honey, Kota Bharu, Malaysia. 13th - 16th 2010 Abstracts: P24

[B13] Nurul Syazana NS Gan SH Halim AS Volatile compositions of Malaysian Tualang (Koompasia Excelsa) honey. FP 13 2nd International Conference on the Medicinal Use of Honey, Kota Bharu, Malaysia. 13th - 16th 2010

[B14] Odeh I Abu-Lafi S Dewik H Al-Najjar I Imam A Valery M A variety of volatile compounds as markers in Palestinian honey from Thymus capitatus, Thymelaea hirsuta, and Tolpis virgata Food Chem 2007 101 4 1393 1397 10.1016/j.foodchem.2006.03.046

[B15] Lušić D Koprivnjak O Ćurić D Sabatini A Volatile profile of Croatian lime tree (Tilia sp.), fir honeydew (Abies alba) and sage (Salvia officinalis) honey Food Technol Biotechno 2007 45 156 165

[B16] Saravana KJ Mandal M Antiproliferative effects of honey and of its polyphenols: A review Journal of Biomedicine and Biotechnology 2009 1 13 10.1155/2009/830616 PMC2712839 19636435

[B17] Nuriza Tumin Arsyayiah N Halim A Shahjahan M Noor Izani N Munavvar J Sattar A Hye Khan Abdul Mohsin SSJ Antibacterial activity of local Malaysian honey Malaysian Journal of Pharmaceutical Sciences 2005 2 1 10

[B18] Snowdon JA Cliver Do Microorganisms in honey, review article Int J Food Microbiol 1996 31 1 26 10.1016/0168-1605(96)00970-1 8880294

[B19] Yusof Norimah Ainul Hafiza AH Zohdi Rozaini M Bakar Zuki A Development of honey hydrogel dressing for enhanced wound healing Radiation Physics and Chemistry 2007 76 1767 1770 10.1016/j.radphyschem.2007.02.107

[B20] Aljady AM Kamaruddin MY Jamal AM Mohd Yassim MY Biochemical study on the efficacy of Malaysian honey on inflicted wounds: an animal model Medical Journal of Islamic Academy of Sciences 2000 13 3 125 132

[B21] Agata KD Ewa SS Robert DW Artur S Rafal S Jerzy P Jerzy S Efficiency assessment of antimicrobial activity of honey-balm on experimental burn wounds Bull Vet Inst Pulawy 2004 48 109 112

[B22] Qualitative and Quantitative Analysis in Microbiology World of Microbiology and Immunology 2003 Encyclopedia.com

[B23] Henry DI Essential procedures for clinical microbiology American Society for Microbiology Press 1998 Blackwell Science

[B24] Suguna I Chandrakasan G Ramamoorthy U Joseph KT Influence of honey on biochemical and biophysical parameters of wounds in rats J Clin Biochem Nutr 1993 14 91 99

[B25] Subrahmanyam M A prospective randomized clinical and histological study of superficial burn wound healing with honey and silver sulfadiazine Burns 1998 24 157 161 10.1016/S0305-4179(97)00113-7 9625243

[B26] Dunford C Cooper R Molan PC White R The use of honey in wound management Nurs Stand 2000 15 63 68 11971572 10.7748/ns2000.11.15.11.63.c2952

[B27] Osuagwu FC Oladejo OW Imosemi IO Enhanced wound contraction in fresh wounds dressed with honey in Wistar rats (Rattus Novergicus) West Afr J Med 2004 23 114 118 15287287 10.4314/wajm.v23i2.28100

[B28] Bergman A Yanai J Weiss J Bell D David MP Acceleration of wound healing by topical application of honey. An animal model Am J Surg 1983 145 3 374 376 10.1016/0002-9610(83)90204-0 6837863

[B29] Molan PC Potential of honey in the treatment of wounds and burns Am J Clin Dermatol 2001 2 13 19 10.2165/00128071-200102010-00003 11702616

[B30] Lusby PE Coombes AL Wilkinson JM Bactericidal activity of different honeys against pathogenic bacteria Arch Med Res 2005 36 464 467 10.1016/j.arcmed.2005.03.038 16099322

[B31] Tan HZ Rosliza AB Gan SH Halim AS Hassan SA Sulaiman SA Kirnpal-Kaur BS The antibacterial properties of Malaysian tualang honey against wound and enteric microorganisms in comparison to manuka honey BMC Complementary and Alternative Medicine 2009 9 34 1 8 19754926 10.1186/1472-6882-9-34 PMC2753561

[B32] Nasir NAM Halim AS Kirnpal-Kaur BS Dorai AA Haneef MNM Antibacterial properties of tualang honey and its effect in burn wound management: a comparative study BMC Complementary and Alternative Medicine 2010 10 31 1 7 20576085 10.1186/1472-6882-10-31 PMC2908556

[B33] Cooper R Molan PC The use of honey as an antiseptic in managing Pseudomonas infection J Wound Care 1999 8 161 164 10455629 10.12968/jowc.1999.8.4.25867

[B34] Al-Waili NS Investigating the antimicrobial activity of natural honey and its effects on the pathogenic bacterial infections of surgical wounds and conjunctiva J Med Food 2004 7 210 222 10.1089/1096620041224139 15298770

[B35] Sharma BR Infection in patients with severe burns: causes and prevention thereof Infect Dis Clin North Am 2007 21 745 759 10.1016/j.idc.2007.06.003 17826621

